# Supplementary material for: SNARE disassembly requires Sec18/NSF side loading
Source: Nat Struct Mol Biol. 2025 Jul 2;32(9):1708–20. doi: 10.1038/s41594-025-01590-w (PMC12440825; doi:10.1038/s41594-025-01590-w)

nature structural & molecular biology

Supplementary information https://doi.org/10.1038/s41594-025-01590-w

SNARE disassembly requires Sec18/NSF side loading

In the format provided by the authors and unedited

Supplementary Information

Sec18 side-loading is essential for universal SNARE recycling across cellular contexts

Yousuf A. Khan[1,2,3,4,9], K. Ian White[1,2,3,4,5], Richard A. Pfuetzner[1,2,3,4,5], Bharti Singal[6],

Luis Esquivies[1,2,3,4,5], Garvey Mckenzie[7], Fang Liu[7], Katherine DeLong[1],

Ucheor B. Choi[1,2,3,4,5], Elizabeth Montabana[6], Theresa Mclaughlin[7], William T. Wickner[8],

Axel T. Brunger[1,2,3,4,5,9]

[1]Department of Molecular and Cellular Physiology, Stanford University, Stanford, CA, USA
[2]Department of Neurology and Neurological Sciences, Stanford University, Stanford, CA, USA
[3]Department of Structural Biology, Stanford University, Stanford, CA, USA
[4]Department of Photon Science, Stanford University, Stanford, CA, USA
[5]Howard Hughes Medical Institute, Stanford University, Stanford, CA, USA
[6]Stanford Cryo-EM microscopy center, Stanford University, Palo Alto, CA, USA
[7]Stanford University Mass Spectrometry, Stanford University, Palo Alto, CA, USA
[8]Department of Biochemistry and Cell Biology, Geisel School of Medicine at Dartmouth, Dartmouth College, Hanover, NH 03755.
[9]Correspondence: yousuf@stanford.edu (YAK), brunger@stanford.edu (ATB)

Table of Contents

Supplementary Information 1
Table of Contents 2
Supplementary discussion 3
Supplementary discussion references 5
Supplementary Figure Legends 6
Supplementary Figure 1 7
Supplementary Figure 2 8
Supplementary Figure 3 9
Supplementary Figure 4 10
Supplementary Figure 5 11
Supplementary Figure 6 12
Supplementary Figure 7 13
Supplementary Figure 8 14

Supplementary discussion

Cross complementation experiments

In our cross-complementation experiments, Sec18 and Sec17 disassembled neuronal and yeast SNARE complexes (**Extended Data Figure 5D-E**), like NSF and α-SNAP disassembling ySNARE. NSF disassembled the ySNARE complex at a rate comparable to Sec18 (**Extended Data Figure 5D**), but, interestingly, Sec18 disassembled neuronal SNARE slower than NSF, ~50-fold slower (**Extended Data Figure 5E**). This cross-complementation activity is corroborated by the high sequence and structural similarities between the yeast and neuronal systems. Despite differences in the SNARE substrate primary sequences, the substrate adopts a similar backbone conformation inside the pore of the D1 ATPase ring in both the yeast and neuronal systems (**Extended Data Figure 4C**).

Variability in N-domain engagement and Sec17 stoichiometry underlies substrate engagement

The eight classes from the non-hydrolyzing y20S dataset also show differences in the ySNARE-Sec17-Sec18-N-domain subcomplex arrangement ("spire") and stoichiometry above the D1 ring of the y20S complex before disassembly (**Figure 2D-E**). Based on an unbiased Cα clustering algorithm (**Methods**), we found that the eight classes fall into four clusters. In cluster 1, classes 1 and 2 with two Sec17 adaptor molecules (the density for the third adaptor is weak) are bound by only three N-domains. In cluster 2, classes 3-5 with a stable, U-shaped formation of three Sec17 molecules are bound by 3 or 4 N-domains. Cluster 3 is defined by a more stable arrangement of the U-shaped Sec17 arrangement again but with an N-domain contacting at least one Sec17 adaptor protein. Lastly, in cluster 4, Y20S class 8 has all five proximal protomers' N-domains engaging the Sec17 adaptor proteins.

Comparing the most extreme class averages in this dataset (class 1 and class 8, **Figure 2D**), the two Sec17 molecules lack the surface area to fully cover the exposed SNARE surface, which is covered upon binding a third Sec17 molecule (note that the linker between the two Sec9 SNAREs prevents a fourth Sec17 molecule from binding). This configuration also leads to a difference in the angle of the spire relative to the ring, where class 1's spire has an acute angle relative to the D1 ring. In contrast, class 8's spire is almost perpendicular. α-SNAP initially binds the SNARE complex with a 1:1 stoichiometry[7], yet structural and single-molecule FRET data shows that the 20S can form with up to 4 α-SNAP molecules[8–11]. Forming a 1:1 complex of SNARE and adaptor allows for Sec18/NSF recognition via N-domain engagement. Then, additional Sec17 molecules bind to the spire before disassembly, allowing for additional connection points between Sec18 and the spire. Regardless of class, the arrangement of the protomers around the SNARE substrate in the D1 ring and the D2 ring arrangement is nearly identical, as the longer N-D1 linker is flexible enough to accommodate a variety of N-domain configurations.

As in the case of α-SNAP, Sec17 interacts with the SNARE domain primarily through electrostatic interactions, consistent with structures of the neuronal 20S complex[8]. Alternating belts of electrostatic surface potentials complement each other in binding Sec17 adaptor proteins to the SNARE ternary complex (**Extended Data Figure 3B**). This observation may

explain how Sec18 and Sec17 can process many SNARE complexes composed of different primary sequences. The N-domains and Sec17p domains have complementary electrostatic surface potentials, suggesting that charge-charge interactions play a role in this interaction. The N-domains form cavities of opposite charge that complement the C-terminal portion of Sec17 upon binding (**Extended Data Figure 3C**).

Vacuolar SNARE fusion assay

Vacuolar SNARE components were also found in our *in vivo* crosslinking experiment (**Figure 1B**), making the vacuolar an attractive candidate for a functional test. The vacuolar proteoliposome fusion assay mimics the homotypic fusion of lysosomes/vacuoles[12] in which membranes expressing sets of SNAREs on each membrane must be preprocessed by Sec18, Sec17 and then passed to the HOPS complex to allow for homotypic fusion to occur. Using our preparation of Sec18, we observed fusion activity, which increased commensurately with the increase in Sec18 concentration. This observation confirmed that vacuolar SNARE processing was occurring, allowing homotypic fusion.

Substrate-free NSF under hydrolyzing conditions reveals additional conformations.

We investigated whether NSF may also form these ring states under hydrolyzing conditions without an SNARE substrate. We purified NSF, exchanged it into a hydrolyzing buffer (*i.e.*, in the presence of $Mg^{2+)}$, and determined cryo-EM structures. We found two primary oligomeric states, consistent with our Sec18 data, a flat heptameric (**Extended Data Figure 7A**) and a hexameric (**Extended Data Figure 7B-D**) state. These states of NSF are like the heptameric and split hexameric states of Sec18, with a flat D1 ring and ADP in all D1 protomers and ATP in all D2 protomers.

The D1 ring of hexameric NSF contains density in the pore (**Extended Data Figure 7B–E**); the most likely interpretation of this density is that NSF is engaged with part of the N-domain from one of the protomers (**Extended Data Figure 7F–G**). This structure suggests that Sec18/NSF tends to fill the side-loading gap in the absence of a SNARE substrate. Taken together, either Sec18/NSF accepts an extra protomer that can easily slide in or out (or binds to its N-domain), or it accepts a SNARE substrate, leading to hexameric 20S or Y20S complex formation. We note that we did not observe a pronounced coordinated ring opening in this condition, although starting from the assembled 20S complex, we did observe a coordinated split-open state of both D1 and D2 rings[18] . Instead, the particular conditions of the substrate-free NSF sample shift it to a self-inhibited hexameric state where the N-terminal domain is engaged with the D1 pore.

Our observations of heptameric or auto-engaged hexameric complexes, also seen in other AAA+ proteins[1,2], follow a broader trend of inactive AAA+ conformations[3–6] that appear reversible in the presence of substrate. We also note that the interaction between protomers differs depending on their oligomeric state (**Supplementary Figure 7**).

Whether or not Sec18/NSF heptamers are present in the cell at physiologically relevant conditions is unclear and will be subject to future investigations.

Supplementary discussion references

1. Shen, P. S. Rearranging AAA+ architecture to accommodate folded substrates. *Nat. Struct. Mol. Biol.* **27**, 225–226 (2020).
2. Schumacher, J., Joly, N., Rappas, M., Zhang, X. & Buck, M. Structures and organisation of AAA+ enhancer binding proteins in transcriptional activation. *J. Struct. Biol.* **156**, 190–199 (2006).
3. Gaubitz, C. *et al.* Cryo-EM structures reveal high-resolution mechanism of a DNA polymerase sliding clamp loader. *eLife* **11**, e74175 (2022).
4. Shin, M. *et al.* Structural basis for distinct operational modes and protease activation in AAA+ protease Lon. *Sci. Adv.* **6**, eaba8404 (2020).
5. Gao, H. *et al.* Cryo-EM structures of human p97 double hexamer capture potentiated ATPase-competent state. *Cell Discov.* **8**, 1–13 (2022).
6. Cho, C. *et al.* Structural basis of nucleosome assembly by the Abo1 AAA+ ATPase histone chaperone. *Nat. Commun.* **10**, 5764 (2019).
7. Vivona, S. *et al.* Disassembly of All SNARE Complexes by N-Ethylmaleimide-sensitive Factor (NSF) Is Initiated by a Conserved 1:1 Interaction between α-Soluble NSF Attachment Protein (SNAP) and SNARE Complex*,. *J. Biol. Chem.* **288**, 24984–24991 (2013).
8. White, K. I., Zhao, M., Choi, U. B., Pfuetzner, R. A. & Brunger, A. T. Structural principles of SNARE complex recognition by the AAA+ protein NSF. *eLife* **7**, e38888 (2018).
9. Zhao, M. *et al.* Mechanistic insights into the recycling machine of the SNARE complex. *Nature* **518**, 61–67 (2015).
10. White, K. I. *et al.* NSF converts syntaxin clusters to a priming-ready state. *Unpublished*.
11. Kim, C. *et al.* Extreme parsimony in ATP consumption by 20S complexes in the global disassembly of single SNARE complexes. *Nat. Commun.* **12**, 3206 (2021).
12. Wickner, W. Membrane fusion: five lipids, four SNAREs, three chaperones, two nucleotides, and a Rab, all dancing in a ring on yeast vacuoles. *Annu. Rev. Cell Dev. Biol.* **26**, 115–136 (2010).
13. DAmico, K. A. *et al.* Structure of a membrane tethering complex incorporating multiple SNAREs. *Nat. Struct. Mol. Biol.* 1–9 (2024) doi:10.1038/s41594-023-01164-8.
14. Shvarev, D. *et al.* Structure of the HOPS tethering complex, a lysosomal membrane fusion machinery. *eLife* **11**, e80901 (2022).
15. Song, H., Orr, A. S., Lee, M., Harner, M. E. & Wickner, W. T. HOPS recognizes each SNARE, assembling ternary trans-complexes for rapid fusion upon engagement with the 4th SNARE. *eLife* **9**, e53559 (2020).
16. Song, H., Torng, T. L., Orr, A. S., Brunger, A. T. & Wickner, W. T. Sec17/Sec18 can support membrane fusion without help from completion of SNARE zippering. *eLife* https://elifesciences.org/articles/67578 (2021) doi:10.7554/eLife.67578.
17. Orr, A. & Wickner, W. Sec18 supports membrane fusion by promoting Sec17 membrane association. *Mol. Biol. Cell* **33**, ar127 (2022).
18. White, K. I. *et al.* NSF converts syntaxin clusters to a priming-ready state. *Unpublished*.

Supplementary Figure Legends

Supplementary Figure 1 | Gene ontology network of enriched processes based on enriched proteins in Sec18 coIP.

Supplementary Figure 2 | Processing information for the cryo-EM datasets. a) The general processing workflow that was employed for each dataset. b) Sec18 substratefree representative micrograph, Class2D, Class3D images, and final refinement depictions. c) Y20S EDTA representative micrograph, Class2D, Class3D images, and final refinement depictions. d) Y20S hydrolyzing condition representative micrograph, Class2D, Class3D images, and final refinement depictions. e) NSF hydrolyzing condition representative micrograph, Class2D, Class3D images, and final refinement depictions.

Supplementary Figure 3 | Distribution of particle orientation, FSC curves, and local resolution for all Y20S EDTA classes.

Supplementary Figure 4 | Distribution of particle orientation, FSC curves, and local resolution for all Y20S hydrolyzing classes.

Supplementary Figure 5 | Distribution of particle orientation, FSC curves, and local resolution for all Sec18 hydrolyzing classes.

Supplementary Figure 6 | Distribution of particle orientation, FSC curves, and local resolution for all NSF hydrolyzing classes.

Supplementary Figure 7 | Interprotomer interface comparison of substrate-engaged (top) and substrate-free (bottom) protomers of Sec18. Interacting residues at the interface are shown.

Supplementary Figure 8 | Full gel image of native gel of Sec18 hydrolyzing no substrate cryoEM sample, corresponding to cropped lanes shown in Extended Data Fig. 6d.

Supplementary Figure 1

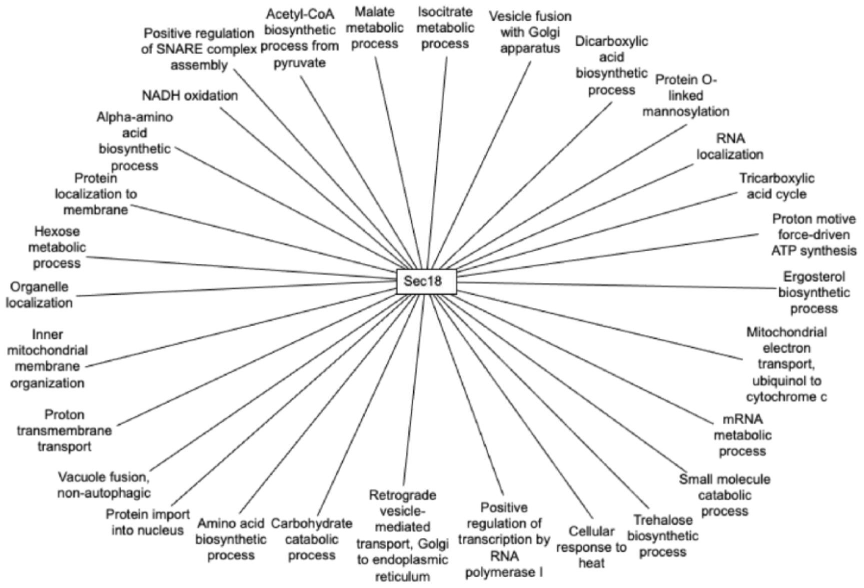

Supplementary Figure 2

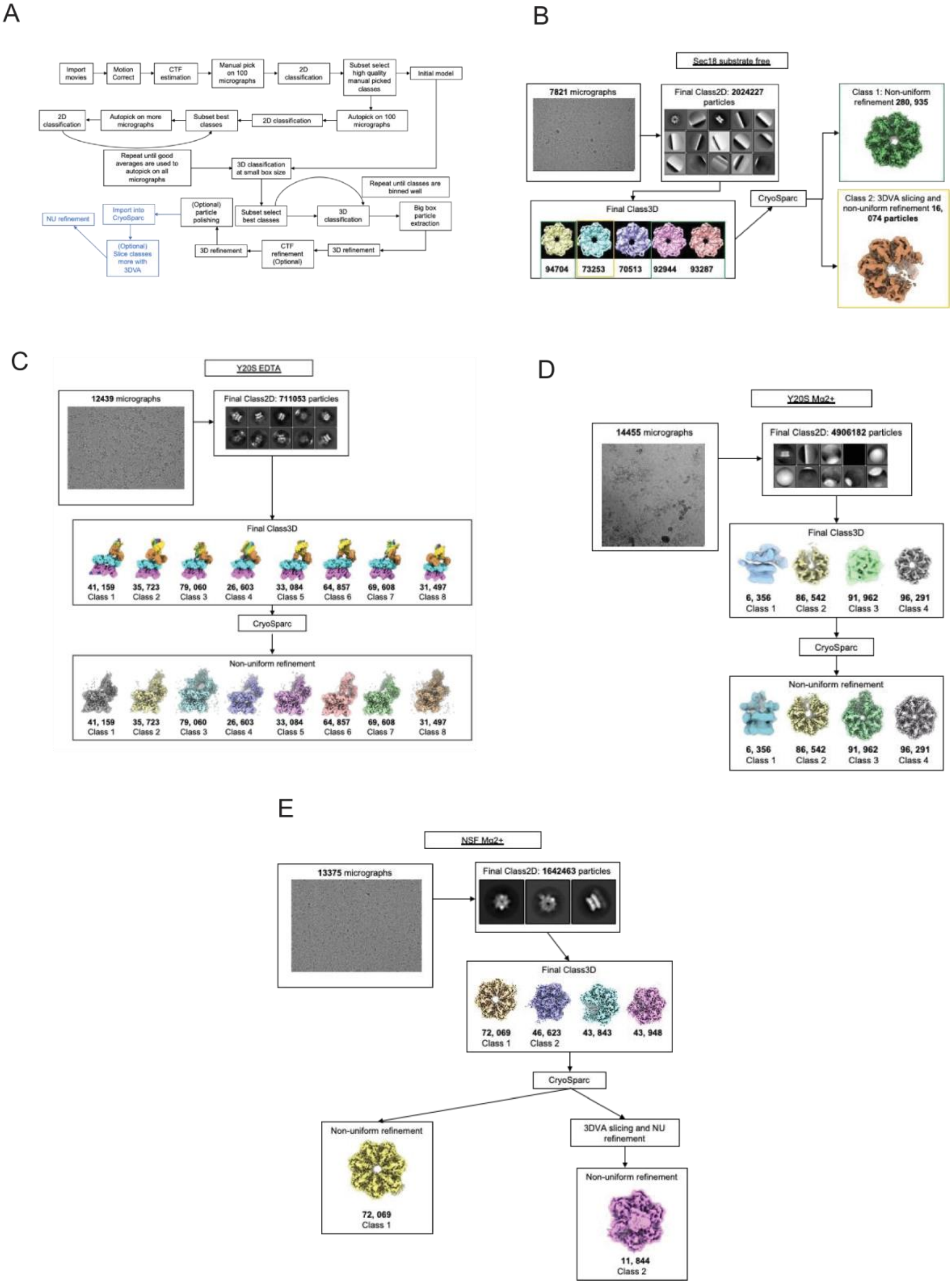

Supplementary Figure 3

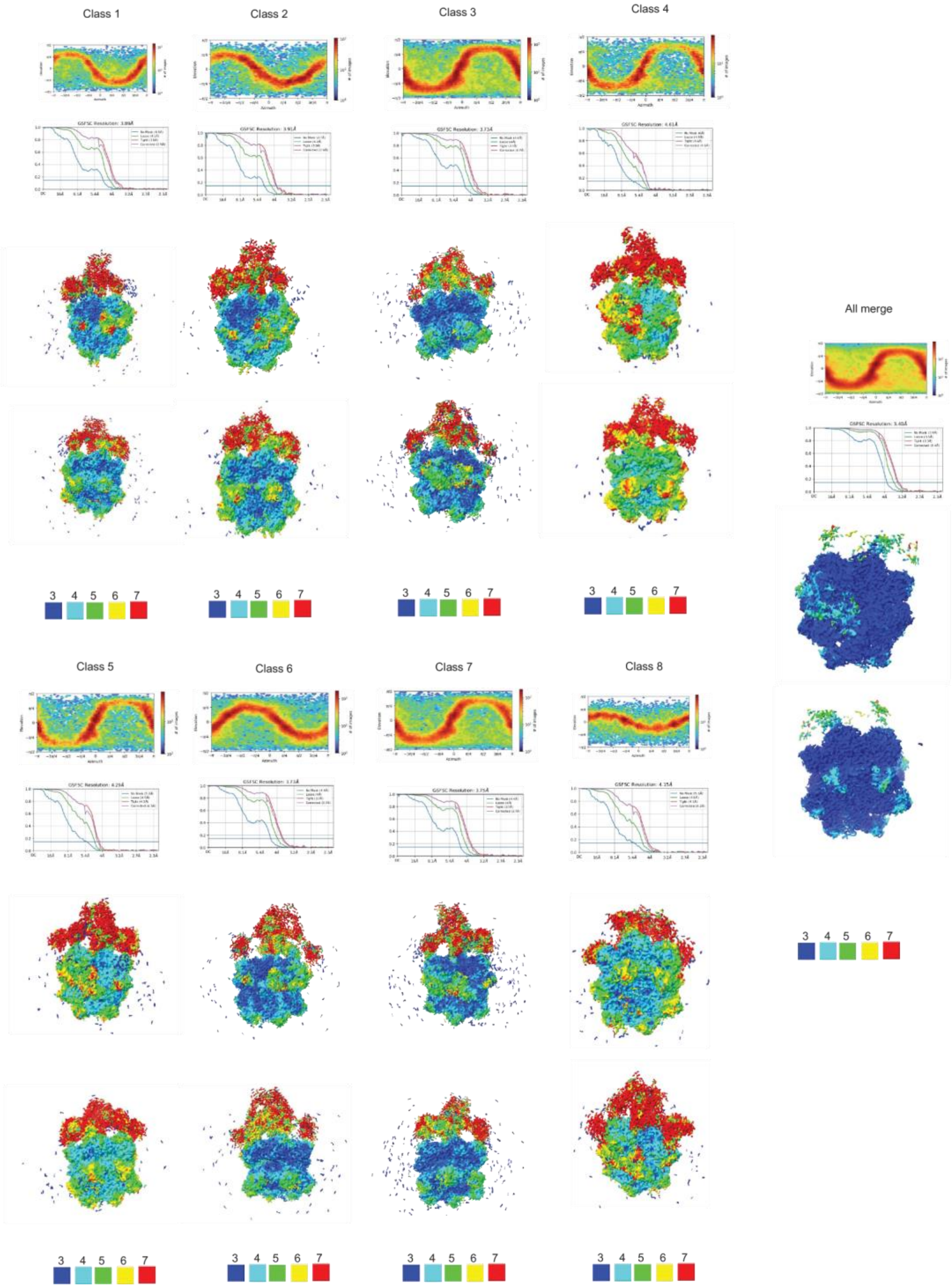

Supplementary Figure 4

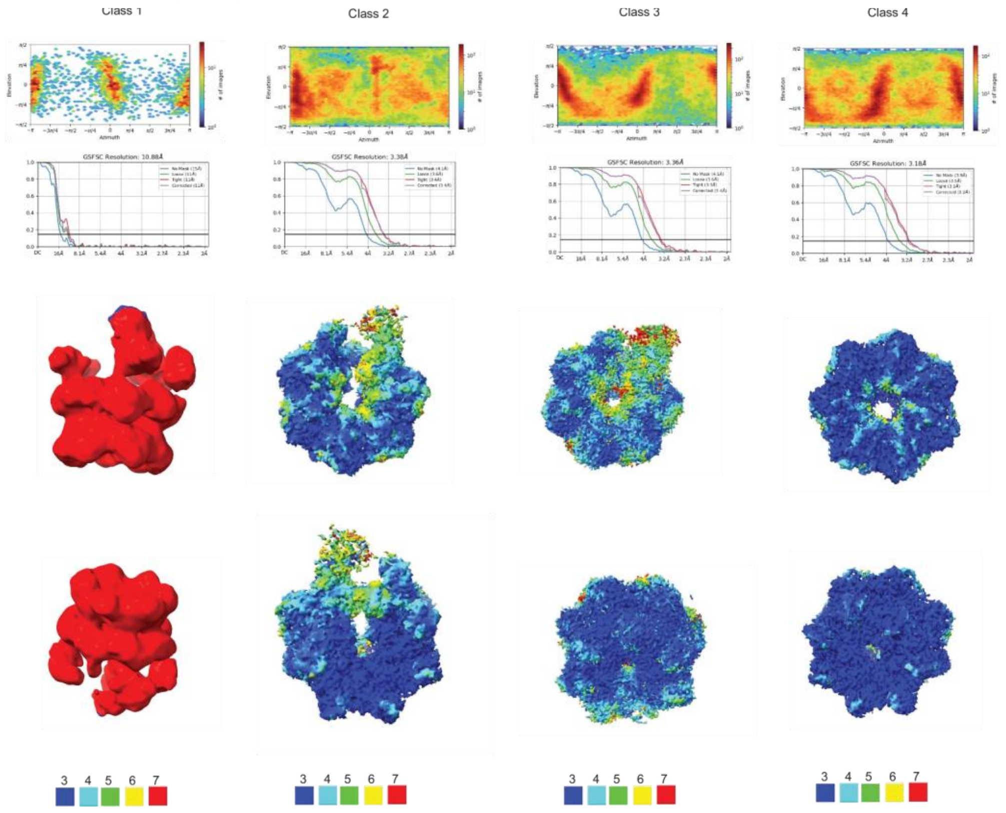

Supplementary Figure 5

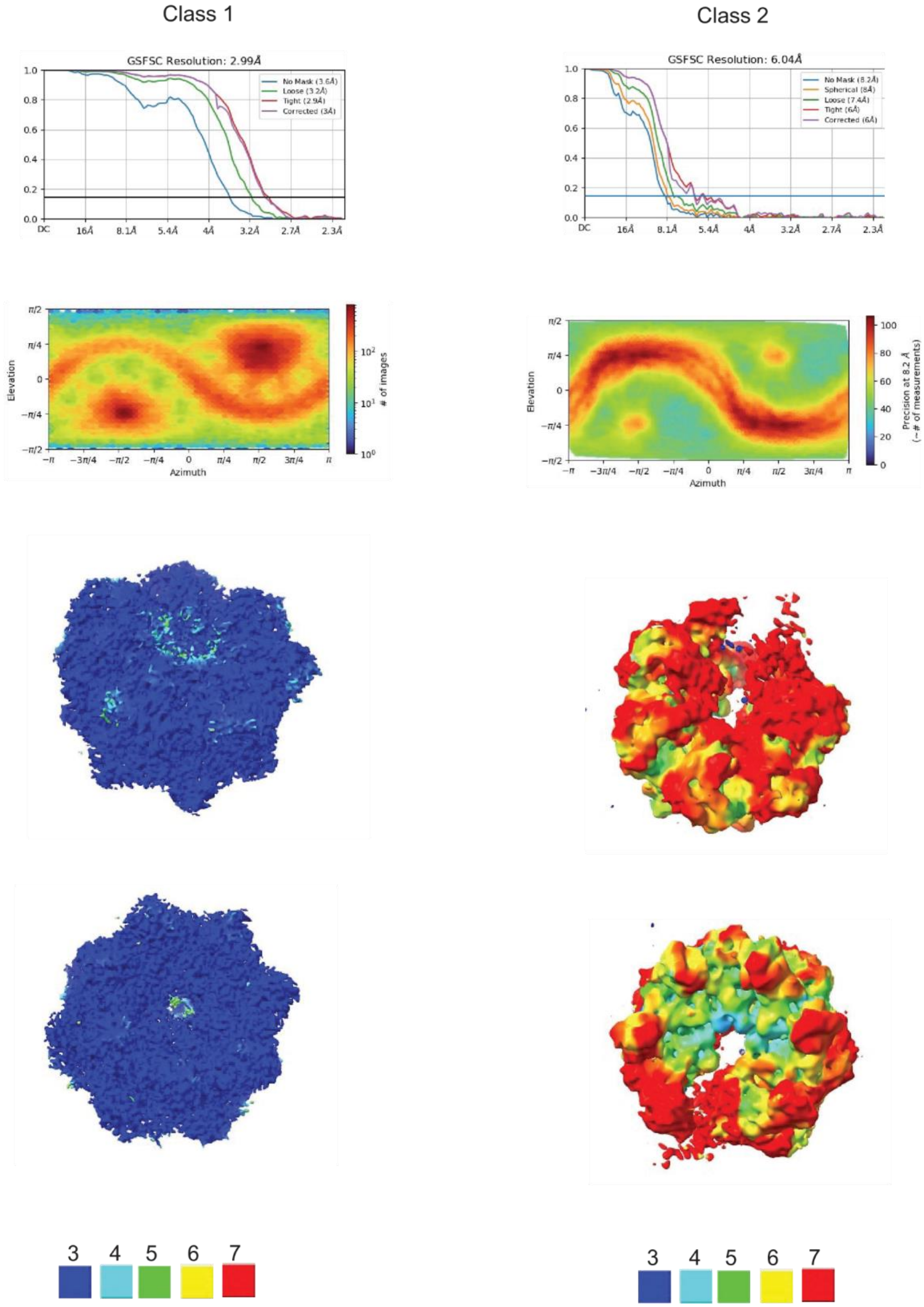

Supplementary Figure 6

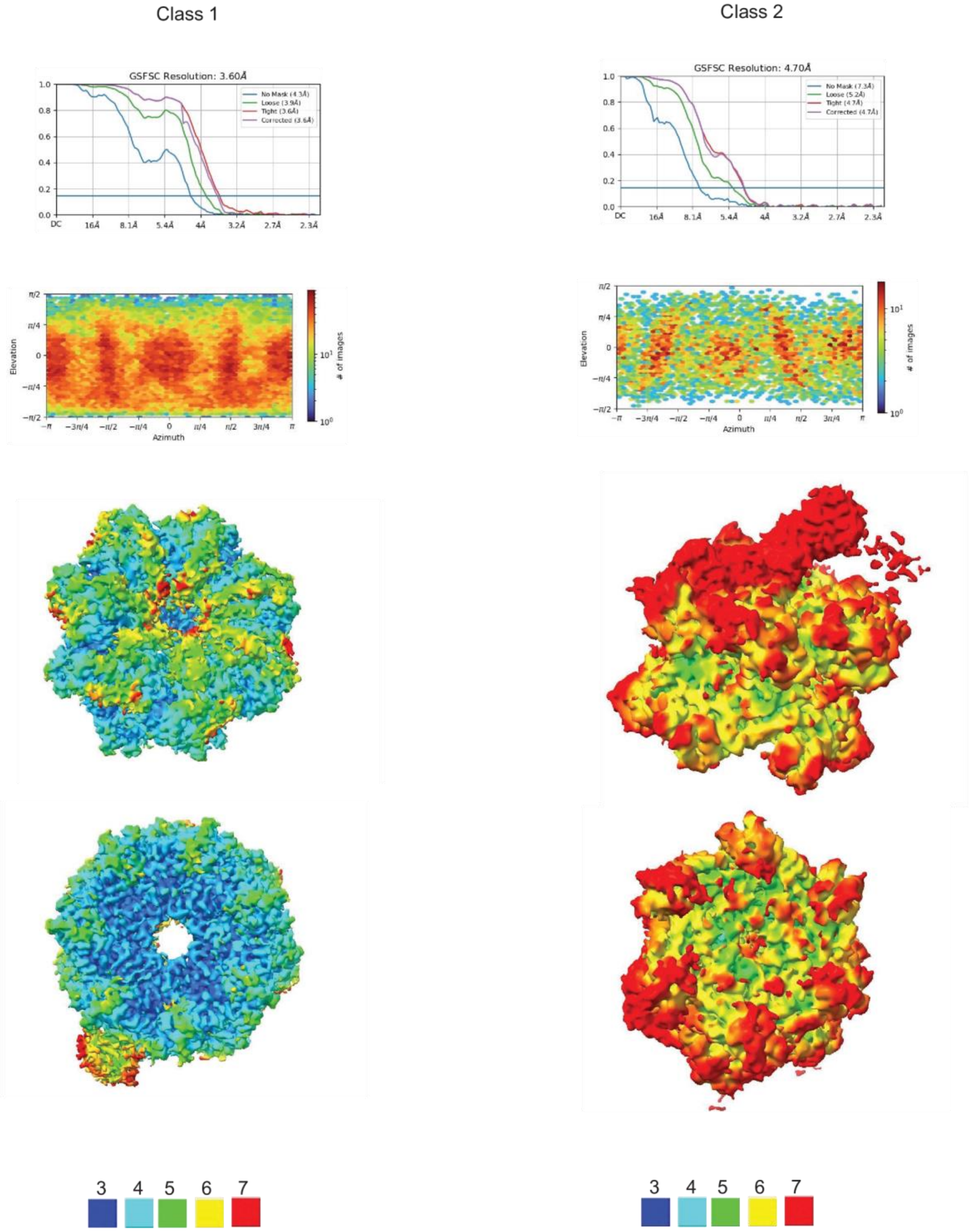

Supplementary Figure 7

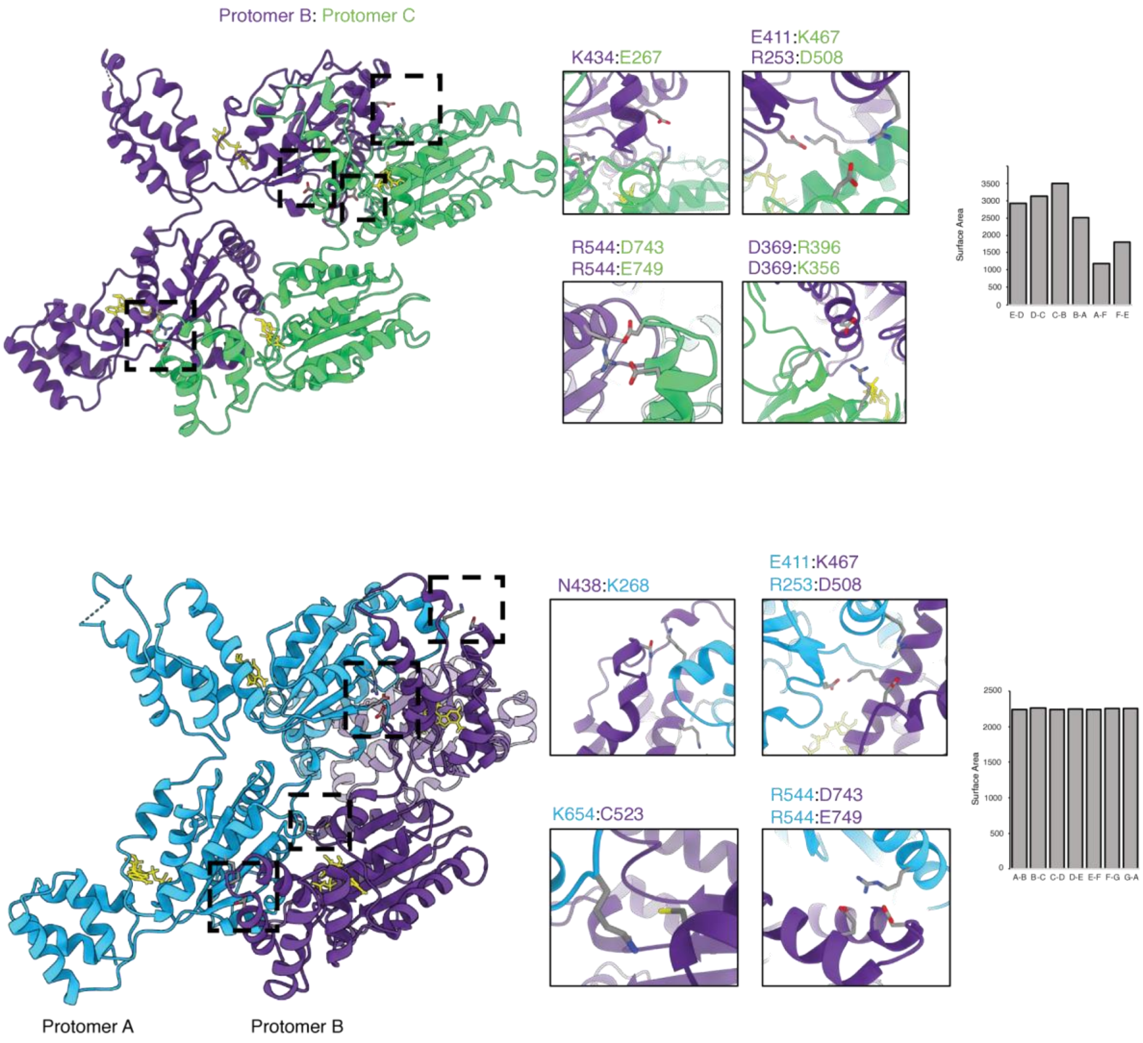

Supplementary Figure 8

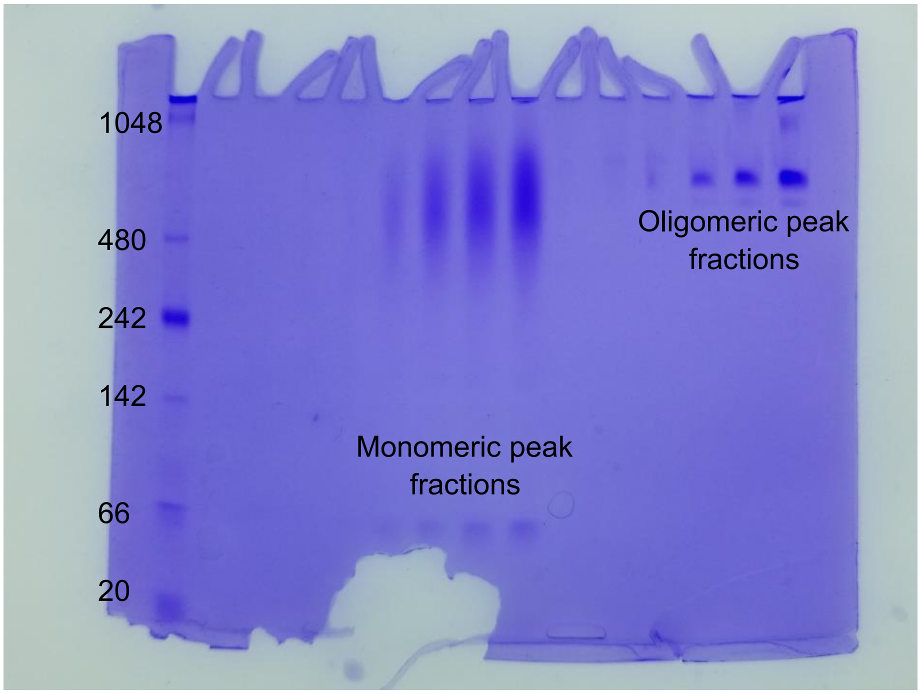

Supplement: Supplementary file 1 — Supplementary Figs. 1–7, Discussion and Gel. [file 41594_2025_1590_MOESM1_ESM.pdf]
