# Supplementary material for: SNARE disassembly requires Sec18/NSF side loading
Source: Nat Struct Mol Biol. 2025 Jul 2;32(9):1708–20. doi: 10.1038/s41594-025-01590-w (PMC12440825; doi:10.1038/s41594-025-01590-w)
Supplement: Supplementary file 2 — Reporting Summary [file 41594_2025_1590_MOESM2_ESM.pdf]

nature portfolio

Corresponding author(s): Axel T. Brunger, Yousuf A. Khan

Last updated by author(s): May 14, 2025

Reporting Summary

Nature Portfolio wishes to improve the reproducibility of the work that we publish. This form provides structure for consistency and transparency in reporting. For further information on Nature Portfolio policies, see our Editorial Policies and the Editorial Policy Checklist.

Statistics

For all statistical analyses, confirm that the following items are present in the figure legend, table legend, main text, or Methods section.

n/a	Confirmed	
☐	☒	The exact sample size (*n*) for each experimental group/condition, given as a discrete number and unit of measurement
☐	☒	A statement on whether measurements were taken from distinct samples or whether the same sample was measured repeatedly
☐	☒	The statistical test(s) used AND whether they are one- or two-sided<br>*Only common tests should be described solely by name; describe more complex techniques in the Methods section.*
☒	☐	A description of all covariates tested
☐	☒	A description of any assumptions or corrections, such as tests of normality and adjustment for multiple comparisons
☐	☒	A full description of the statistical parameters including central tendency (e.g. means) or other basic estimates (e.g. regression coefficient) AND variation (e.g. standard deviation) or associated estimates of uncertainty (e.g. confidence intervals)
☐	☒	For null hypothesis testing, the test statistic (e.g. F, t, r) with confidence intervals, effect sizes, degrees of freedom and P value noted<br>*Give P values as exact values whenever suitable.*
☒	☐	For Bayesian analysis, information on the choice of priors and Markov chain Monte Carlo settings
☒	☐	For hierarchical and complex designs, identification of the appropriate level for tests and full reporting of outcomes
☒	☐	Estimates of effect sizes (e.g. Cohen's *d*, Pearson's *r*), indicating how they were calculated

Our web collection on statistics for biologists contains articles on many of the points above.

Software and code

Policy information about availability of computer code

Data collection	SerialEM 4.1, Molecular Devices SoftMax Pro
Data analysis	Tmaven, Byonic v5.x.x, AlphaFold2, ChimeraX 1.8, Coot 0.9, CryoSPARC 4.X, CTFFIND4, ImageJ, ISOLDE, MolProbity, MotionCorr2, PHENIX 1.2.X, GraphPad Prism, RELION 3, custom Python 3.X scripts using packages numpy, scipy, scikit-learn, biopython Bio.PDB, pandas, and matplotlib.

For manuscripts utilizing custom algorithms or software that are central to the research but not yet described in published literature, software must be made available to editors and reviewers. We strongly encourage code deposition in a community repository (e.g. GitHub). See the Nature Portfolio guidelines for submitting code & software for further information.

Data

Policy information about availability of data

All manuscripts must include a data availability statement. This statement should provide the following information, where applicable:

- Accession codes, unique identifiers, or web links for publicly available datasets
- A description of any restrictions on data availability
- For clinical datasets or third party data, please ensure that the statement adheres to our policy

Accession codes available upon publication. No restrictions on data availability.

Research involving human participants, their data, or biological material

Policy information about studies with human participants or human data. See also policy information about sex, gender (identity/presentation), and sexual orientation and race, ethnicity and racism.

Reporting on sex and gender	Does not apply
Reporting on race, ethnicity, or other socially relevant groupings	Does not apply
Population characteristics	Does not apply
Recruitment	Does not apply
Ethics oversight	Does not apply

Note that full information on the approval of the study protocol must also be provided in the manuscript.

Field-specific reporting

Please select the one below that is the best fit for your research. If you are not sure, read the appropriate sections before making your selection.

☒ Life sciences ☐ Behavioural & social sciences ☐ Ecological, evolutionary & environmental sciences

For a reference copy of the document with all sections, see nature.com/documents/nr-reporting-summary-flat.pdf

Life sciences study design

All studies must disclose on these points even when the disclosure is negative.

Sample size	Disassembly assays described in this study were performed with at least 3 samples for each group, which is consistent with sample sizes commonly used in similar studies in the field. Single molecule FRET assays have sample size (number of traces analyzed) for each condition in main figure panels.
Data exclusions	Cryo-EM reconstructions with resolutions worse than 9 Å were excluded due to challenges associated with accurate model building and subsequent comparison with other reconstructions. For single molecule FRET experiments, traces were automatically excluded prior to analysis by automated tMaven filtering that had no manual intervention or parameter tuning.
Replication	In-vitro assays were performed in different batches with different mixes of in-vitro proteins to ensure independence of each assay. For in-vivo mass-spec, uninduced and induced samples were always processed simultaneously and only differed by galactose induction in cell media prior to processing.
Randomization	N/A
Blinding	N/A

Reporting for specific materials, systems and methods

We require information from authors about some types of materials, experimental systems and methods used in many studies. Here, indicate whether each material, system or method listed is relevant to your study. If you are not sure if a list item applies to your research, read the appropriate section before selecting a response.

Materials & experimental systems

n/a	Involved in the study	
☐	☒	Antibodies
☐	☒	Eukaryotic cell lines
☒	☐	Palaeontology and archaeology
☒	☐	Animals and other organisms
☒	☐	Clinical data
☒	☐	Dual use research of concern
☒	☐	Plants

Methods

n/a	Involved in the study	
☒	☐	ChIP-seq
☒	☐	Flow cytometry
☒	☐	MRI-based neuroimaging

Antibodies

Antibodies used	Pierce HA-Tag IP/Co-IP kit
Validation	IP of HA-tagged SRF and HA-tagged Pak1 were performed. For HA-Sec18, IP was confirmed independently by western blot with a different HA antibody to confirm enrichment of HA-Sec18.

Eukaryotic cell lines

Policy information about cell lines and Sex and Gender in Research

Cell line source(s)	S288C Saccharomyces cerevisiae
Authentication	18S ribosomal RNA sequencing and 28S ribosomal RNA sequencing
Mycoplasma contamination	N/A
Commonly misidentified lines (See ICLAC register)	N/A

Plants

Seed stocks	N/A
Novel plant genotypes	N/A
Authentication	N/A
